# Role of Plasma Membrane Caveolae/Lipid Rafts in VEGF-Induced Redox Signaling in Human Leukemia Cells

**DOI:** 10.1155/2014/857504

**Published:** 2014-03-11

**Authors:** Cristiana Caliceti, Laura Zambonin, Benedetta Rizzo, Diana Fiorentini, Francesco Vieceli Dalla Sega, Silvana Hrelia, Cecilia Prata

**Affiliations:** ^1^Department of Clinical and Experimental Medicine, University of Ferrara, Via Fossato di Mortara 66, 44121 Ferrara, Italy; ^2^Department of Pharmacy and Biotechnology, Alma Mater Studiorum-University of Bologna, Via Irnerio 48, 40126 Bologna, Italy; ^3^Department for Life Quality Studies, Alma Mater Studiorum-University of Bologna, C.so Augusto 237, 47921 Rimini, Italy

## Abstract

Caveolae/lipid rafts are membrane-rich cholesterol domains endowed with several functions in signal transduction and caveolin-1 (Cav-1) has been reported to be implicated in regulating multiple cancer-associated processes, ranging from tumor growth to multidrug resistance and angiogenesis. Vascular endothelial growth factor receptor-2 (VEGFR-2) and Cav-1 are frequently colocalized, suggesting an important role played by this interaction on cancer cell survival and proliferation. Thus, our attention was directed to a leukemia cell line (B1647) that constitutively produces VEGF and expresses the tyrosine-kinase receptor VEGFR-2. We investigated the presence of VEGFR-2 in caveolae/lipid rafts, focusing on the correlation between reactive oxygen species (ROS) production and glucose transport modulation induced by VEGF, peculiar features of tumor proliferation. In order to better understand the involvement of VEGF/VEGFR-2 in the redox signal transduction, we evaluated the effect of different compounds able to inhibit VEGF interaction with its receptor by different mechanisms, corroborating the obtained results by immunoprecipitation and fluorescence techniques. Results here reported showed that, in B1647 leukemia cells, VEGFR-2 is present in caveolae through association with Cav-1, demonstrating that caveolae/lipid rafts act as platforms for negative modulation of VEGF redox signal transduction cascades leading to glucose uptake and cell proliferation, suggesting therefore novel potential targets.

## 1. Introduction

Caveolae and lipid rafts are ordered structures of membrane microdomains, characterized by high concentration of cholesterol and glycosphingolipids, which are involved in fundamental cellular functions such as endocytosis, protein trafficking, and signal transduction [1–4]. Several different mechanisms probably underlie the lipid raft-controlled cell signaling. For example, rafts may contain incomplete signaling pathways that are activated when a receptor and/or other required molecules are recruited into the raft. Otherwise, rafts may be important in limiting signaling, either by physical sequestration of signaling components to block nonspecific interactions or by suppressing the intrinsic activity of signaling proteins present within rafts [5, 6].

The maintenance of cholesterol levels is essential for functional caveolae and depends, in part, on the interaction of cholesterol with caveolin-1 (Cav-1), the major structural component of caveolae [7]. Various receptors and signaling molecules are localized in these membrane regions and are negatively regulated by Cav-1 through its scaffolding domain. In cancers, it is increasingly clear that Cav-1 is implicated in regulating multiple cancer-associated processes, ranging from cellular transformation, tumor growth, invasion, and metastasis to multidrug resistance and angiogenesis [8, 9].

Recent experimental evidence shows also that Vascular Endothelial Growth Factors (VEGF) promote the release of Vascular Endothelial Growth Factors Receptor 2 (VEGFR-2 or KDR) from caveolae/lipid rafts and its consequent activation, possibly stimulating NAD(P)H oxidases (Nox) in endothelial cells [10]. In these cells, VEGFR-2 is present in caveolae through association with Cav-1, which negatively regulates receptor activity in basal state. Dissociation of VEGFR-2 from caveolae/Cav-1 seems to be essential for VEGFR-2 autophosphorylation and activation of downstream signaling events [7].

There is now a consensus that VEGF family is crucial for vascular development and neovascularisation in both physiologic and pathologic processes. Although both VEGFR-1 and VEGFR-2 are expressed in the vascular endothelium, the angiogenic activities of VEGFs (in particular, VEGF-A) are transduced mainly through VEGFR-2 [11]. VEGF receptors, indeed, are not exclusively expressed by endothelial cells but are also present in hematopoietic cells. This is not a surprising event because during embryonic development, hematopoietic and early endothelial cells (angioblasts) originate from a common precursor known as hemangioblast. Given this common root, several pathways are shared by hematopoietic and vascular cells [12, 13]. Moreover, leukemia cells have been associated with angiogenesis, upon the demonstration that leukemia progression is accompanied by an increase in bone marrow vascularization [14]. In many leukemia cells, VEGF/VEGFR interactions may stimulate proliferation, migration, and survival by autocrine and paracrine loops [15]. In recent studies, the expression of VEGF/VEGFR in acute myeloid leukemia (AML) patients has been detected and the increased levels of plasma VEGF have been correlated with reduced survival and lower remission rates [16, 17].

Therefore, the elucidation of the mechanisms underlying VEGF/VEGFR activity in leukemia cells is necessary for the development of agents to be used in combination with/instead of standard chemotherapy. To achieve this goal, it must be taken into consideration also the strong role played by the redox environment in leukemia survival, growth, progression, relapse, and drug resistance. Reactive oxygen species (ROS), indeed, play both positive and negative roles in cellular proliferation and survival; this feature has been exploited by leukemia cells to promote the hallmarks of cancer phenotype, either through phosphorylation events or transcriptional alteration [18].

In this context, our attention has been focused on the study of the correlation between caveolae/lipid rafts and redox signaling in the human erythromegakaryocytic cell line, B1647, a model of acute myeloid leukemia (AML) constitutively producing VEGF and expressing its tyrosine-kinase receptor VEGFR-2 [19]. We previously showed that Nox-derived ROS are involved in sustaining the high glucose uptake observed in B1647 cells [20]; subsequently, we demonstrated that VEGF-induced ROS derived from Nox2 and Nox4 play a prosurvival role in the leukemia cell line [21].

In the present work, we investigated the potential involvement of plasma membrane caveolae/lipid rafts in VEGF-mediated redox signaling in the human leukemia cell line.

To this purpose, we evaluated the effect of methyl-*β*-cyclodextrin (CD), the most efficient compound used to induce cholesterol depletion from plasma membrane thus disrupting caveolae/lipid rafts [22] on VEGFR-2 distribution in the plasma membrane and on VEGF/VEGFR-2 interaction. Subsequently, the modulation of ROS generation and glucose transporter 1 (Glut1) activity in B1647 cells was investigated.

## 2. Materials and Methods

### 2.1. Chemicals

Iscove's modified Dulbecco's medium (IMDM) was purchased from BioWhittaker (Walkersville, MD, USA), and human serum (HS) was from CambrexBioscience. VEGF was from BioVision (Mountain View, CA, USA). Methyl-*β*-cyclodextrin (CD), diphenyleneiodonium chloride (DPI), phloretin, 2-deoxy-D-glucose (DOG), cholesterol, phenylmethylsulfonyl fluoride (PMSF), N-tosyl-L-lysine chloromethyl ketone (TLCK), N-tosyl-L-phenylalanine chloromethyl ketone (TPCK), sodium orthovanadate, protease inhibitor cocktail, Trypan blue solution (0,4%), 3-(4,5-dimethylthiazol-2-yl)-2,5-diphenyl tetrazolium bromide (MTT), Igepal, Triton X-100, sucrose, Semaxinib (SU5416), mouse monoclonal antiserum against tubulin (no. T7816), and rabbit antibody against flotillin-2 (no. F1805) were from Sigma-Aldrich (St. Louis, MO, USA). 2-Deoxy-D-[2,6-^3^H]-glucose and [1,2-^3^H(N)]-cholesterol were from PerkinElmer (Massachusetts, USA); nitrocellulose paper and ECL Plus Western Blotting Detection Reagents were from GE Healthcare (UK). Bevacizumab was provided by Roche. Triton X-100 and sucrose were from Merck (Whitehouse Station, NJ, USA). DC Protein Assay Kit was from Bio-Rad (USA). Anti-caveolin-1 (no. 610059) and antitransferrin receptor (CD71) (no. 612124) were provided by BD Biosciences (San Jose, CA, USA); anti-Lyn antibody (no. ab1890) was from Abcam (Cambridge, UK). Anti-Glut1 (no. CBL242), anti-VEGF receptor-2 (no. 05-554), and anti-P-tyrosine (no. 06-427) were from Millipore (Temecula, CA, USA). Anti-Glut1 (N-20) (no. sc-1603) and fluorescent FITC-conjugated anti-goat IgG (no. sc-2024) were from Santa Cruz Biotechnology (Santa Cruz, CA, USA). Sulfosuccinimidyl 6-(biotinamido) hexanoate (NHS-LC-biotin) and streptavidin-agarose beads were purchased from Pierce (Rockford, IL, USA).

All the other chemicals and solvents were of the highest analytical grade.

### 2.2. Cell Culture

B1647 is a humane acute myeloid leukemia (AML) cell line cultured in IMDM supplemented with 5% human serum (HS).

The experimental model employed 16–18 h serum-depleted cells, as these conditions were more apt for focusing experiments on self-produced VEGF and cholesterol roles, ruling out other growth factor effects.

### 2.3. Cell Viability Evaluation

Viable cells were evaluated by the Trypan blue exclusion test. Cell viability was also assayed by the MTT assay [23], since the reduction of tetrazolium salts is widely accepted as a reliable way to examine cell viability/proliferation. Cells were incubated with 0.5 mg/mL MTT for 4 h at 37°C. At the end of the incubation, purple formazan salt crystals were formed and dissolved by adding the solubilization solution (10% SDS, 0.01 M HCl) and then the plates were incubated overnight in humidified atmosphere (37°C, 5% CO_2_). The absorption at 570 nm was measured on a multiwell plate reader (Wallac Victor^2^, PerkinElmer).

### 2.4. Cholesterol Depletion

B1647 cells suspended in culture medium were incubated overnight with [^3^H]-cholesterol (0.5 *μ*Ci/mL) and then washed, suspended in PBS, and exposed to different concentrations of CD (5 mM) for different time points (0–30 min). To measure the relative cholesterol content, cells were washed twice in PBS and pelleted at 4.000 g for 1 min and sample radioactivity was quantified by liquid-scintillation counting [24].

### 2.5. Isolation of Membrane Caveolae/Lipid Rafts

Caveolae/lipid rafts and detergent-soluble proteins were separated by flotation assays adapted from previously described methods [24, 25]. 200 × 10^6^ B1647 cells (approximately 6 mg of protein) were washed twice with PBS, pelleted at 300 g for 7 min, and left on ice for 10 min. The cell pellet was incubated at 4°C in 1.2 mL of lysis buffer (1% Triton X-100, 150 mM NaCl, 50 mM TRIS, and 5 mM EDTA supplemented with 0.1 mM PMSF, 0.1 mM TLCK, 0.1 mM TPCK, 1 mM orthovanadate, and protease inhibitor cocktail, pH 8.0). In all subsequent steps, solutions and samples were kept at 4°C. The lysates were then spun for 10 min at 6.000 g in an Eppendorf Microfuge and supernatants were homogenized in a Potter homogenizer with 20 strokes. For sucrose gradient separations, 1.0 mL of 80% sucrose prepared in PBS was mixed with an equal volume of homogenized sample and then overlaid with a 5–40% sucrose linear step gradient (1.3 mL each of 5%, 30%, and 40% sucrose in PBS). After centrifugation in a SW50.1 Beckman rotor at 160.000 g for 18 h at 4°C, nine 500 *μ*L fractions were collected from the top of the gradient. Same volume aliquots of each fraction were added with Laemmli buffer containing both mercaptoethanol and bromophenol blue and boiled for 3 min. Samples were then subjected to SDS-PAGE and immunoblotting.

To measure the relative cholesterol content along the sucrose gradient fractionation, B1647 cells were preincubated at 37°C for 16 hours with [^3^H]-cholesterol (0.1 *μ*Ci/mL) in cell culture medium. Cells exposed (or not) to 5 mM CD for 20 min were lysed with TX-100 at 4°C and subjected to sucrose gradient centrifugation as previously described. [^3^H]-cholesterol content of each of the nine fractions collected was quantified by liquid scintillation counting.

### 2.6. Protein Assay

Protein concentration was usually determined by the Bradford method with BSA as standard [26]. The protein content of fractions obtained from sucrose gradient was determined by a Bio-Rad DC protein assay kit, using BSA in the presence of appropriate concentration of Triton X-100 or SDS as a standard.

### 2.7. Measurement of Intracellular ROS Levels

ROS intracellular level was evaluated using a fluorescent method, implying the probe, 2′,7′-dichlorofluorescin diacetate (DCFH-DA). Briefly, cells were incubated with 5 *μ*M DCFH-DA for 30 min at 37°C and analyzed spectrofluorimetrically at *λ*_Ex/Em_ 485/535 nm in a multiwell plate reader (Wallac Victor^2^, PerkinElmer). Fluorescence values represent the percentage of inhibition of intracellular ROS with respect to controls.

### 2.8. Glucose Transport Assay

The measurement of glucose transport rate was performed according to [20]. In brief, 4 × 10^6^ cells/mL were suspended in PBS, incubated with different* stimuli* and/or inhibitors at 37°C and then treated with a mixture of 2-deoxy-D-[2,6-^3^H] glucose (0.4 *μ*Ci/assay) and 1 mM unlabelled glucose analogue (DOG mixture) for 2 min at 37°C under conditions where the uptake was linear at least for 20 min. After this time, the uptake was stopped by adding phloretin (final concentration, 0.2 mM), a potent inhibitor of glucose transport activity. Sample radioactivity was measured by liquid scintillation counting.

Transported 2-deoxy-D-glucose was less than 20% of the extracellular-sugar concentration; therefore, glucose transport assay could be considered in* zero-trans* conditions [27]. B1647 cells deprived of medium components and suspended in PBS during glucose transport measurements maintained their viability up to 2 hours at 37°C; thus, the number of viable cells during time intervals of experiments was considered constant (data not shown).

To test the effect of CD on the glucose transport activity, cells were incubated at 37°C with 5 mM CD for 20 min, washed, resuspended in 0.5 mL of PBS, and added with DOG mixture for the measurement of glucose uptake as previously described.

### 2.9. SDS-PAGE and Western Blot Analysis

Cells were lysed with buffer (1% Igepal, 150 mM NaCl, 50 mM Tris-HCl, 5 mM EDTA, 0,1 mM PMSF, 0,1 mM TLCK, 0,1 mM TPCK, 1 mM orthovanadate, and protease inhibitor cocktail, pH 8.0) in ice for 15 min. Cell lysates or fractions obtained after sucrose gradient centrifugation were separated on 10% SDS-polyacrylamide gel using a Mini-Protean II apparatus (BioRad Laboratories) and then transferred electrophoretically to nitrocellulose membranes. Nonspecific binding to membrane was blocked by incubating in Tris-buffered saline (TBS)/Tween, pH 8.0, containing 5% nonfat dried milk for 1 hour at room temperature. Blots were probed overnight at 4°C with primary antibodies, washed with TBS/Tween, and then incubated for 1 hour at room temperature with secondary horseradish peroxidase conjugates antibodies. Membranes were washed and the antigens were then visualized by addition of ECL Plus Western Blotting Detection Reagents.

### 2.10. Immunoprecipitation

B1647 cells maintained in the presence or absence of serum (+HS, −HS) for 16 h were lysates as described above for Western Blot. Lysates containing equal protein amounts were incubated overnight with anti-VEGFR-2 or anti-caveolin-1 antibodies. Then, samples were incubated with protein A-Sepharose beads for 1.5 h at 4°C and then pelleted. Pellets were washed four times with lysis buffer, treated with reducing buffer containing 4% 2-mercaptoethanol, and then boiled for 3 min. Samples were then subjected to SDS-PAGE and immunoblotting with primary antibodies (anti-caveolin-1, anti-P-tyrosine, anti-VEGFR-2, and anti-Nox2) washed with TBS/Tween and then incubated for 1 hour at room temperature with the proper secondary horseradish peroxidase conjugate antibodies. Membranes were washed and the antigens were then visualized by addition of ECL Plus Western Blotting Detection Reagents.

### 2.11. Immunofluorescence

B1647 cells (2 × 10^6^) were incubated for 20 min with or without 10 mM CD and then pelleted and fixed in 3% (w/v) paraformaldehyde for 15 min. Cells were washed twice with HBSS, blocked with PBS/BSA 1% (w/v) for 1 hour, and then incubated for 1 hour with 20 *μ*g/mL of anti-Glut1 raised against a peptide within an extracellular domain of the human transporter protein. Cells were then treated for 1 hour with fluorescent FITC-conjugated rabbit anti-goat IgG, mounted on slides, and visualized using an Olympus IX50 microscope.

### 2.12. Statistical Analysis

Results are expressed as means with standard deviation. Differences between the means were determined by two-tailed Student's *t*-test or by Newman-Keuls multiple comparison test following one-way ANOVA and were considered significant at *P* < 0.05.

## 3. Results

### 3.1. Plasma Membrane Cholesterol Depletion by Means of Methyl-*β*-cyclodextrin (CD): Setting of Conditions

Cyclodextrins are cyclic oligomers of glucose that have the capacity to sequester lipophilic molecules in their hydrophobic core [28]. It has been shown that *β*-cyclodextrins remove cholesterol from cultured cells [29–31], and, among the different dextrin derivatives, methyl-*β*-cyclodextrin (CD) was shown to be the most efficient as acceptor of cellular cholesterol and the most commonly used [22, 31]. Therefore, we chose methyl-*β*-cyclodextrin to induce cholesterol depletion from plasma membrane of B1647 cell line and performed experiments to set the desired conditions, as the degree of cholesterol depletion is a function of the *β*-cyclodextrin derivative concentration, incubation time, temperature, and cell type subjected to the treatment [32].

**Figure 1:**
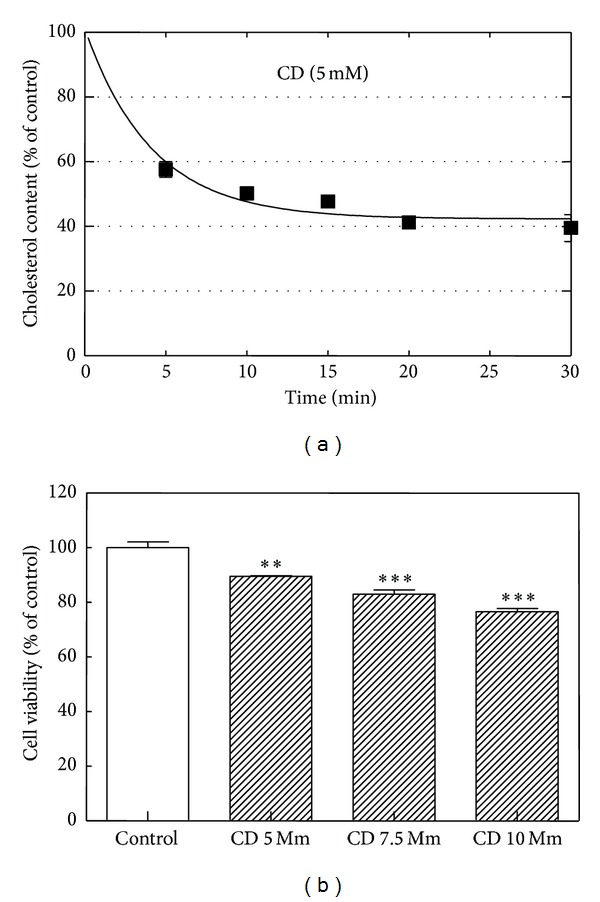
*Effect of incubation time of CD on cholesterol content and cell viability in B1647 cells. *(a) Cells were incubated with [^3^H]-cholesterol (0.5 *μ*Ci/mL) in cell culture medium for 16 h at 37°C, washed, resuspended in PBS, and treated with 5 mM CD for different time periods (0–30 min). Cell suspensions were washed with PBS and then [^3^H]-cholesterol content was estimated by liquid scintillation counting. (b) The viability of the cells treated at different CD concentrations (5–10 mM) for 20 min was evaluated by Trypan Blue exclusion test as described in Section 2. Results are expressed as means ± SD of three independent experiments, each performed in triplicate. **P < 0.005: significantly different from control cells; ***P < 0.0005: significantly different from control cells.

First of all, B1647 cells suspended in culture medium were incubated overnight with [^3^H]-cholesterol (0.5 mCi/mL) and then washed and exposed to different concentrations of CD (2.5–25 mM) for 20–40 min in order to find the best working conditions (not shown).  Figure 1(a) represents the time course of CD effect on cholesterol level, evidencing that 5 mM CD for 20 min was able to remove about 60% cholesterol, keeping cell viability only about 10% decreased, as resulted by Trypan Blue exclusion test (Figure 1(b)). MTT assay was performed under the same conditions, confirming these results (not shown). These conditions, establishing a lipid environment alteration associated with membrane integrity, were selected for the following experiments.

### 3.2. Isolation and Identification of Membrane Caveolae/Lipid Rafts by Detergent Extraction and Sucrose Gradient Centrifugation in B1647 Cell Line

The isolation of membrane caveolae/lipid rafts relies on the relative insolubility in Triton X-100 (TX-100) detergent of membrane regions enriched in cholesterol and glycosphingolipids.

Thus, B1647 cells were lysed using TX-100 and centrifuged and the supernatant was collected and subsequently subjected to sucrose density-gradient (5–40%) centrifugation as described in Section 2. Nine fractions were collected and analyzed by SDS-PAGE followed by Western blotting using antiflotillin-2 (48 kDa), anti-caveolin-1 (24 kDa), and anti-Lyn (58 kDa) antibodies as protein markers for detergent resistant membrane fractions. Indeed, flotillin-2 and Lyn are known to be associated with caveolae/lipid rafts in different cell lines [33], as well as Cav-1, the major structural component of caveolae. The transferrin receptor (CD71), an integral membrane protein, was selected as a marker for nonraft membrane fractions.

**Figure 2:**
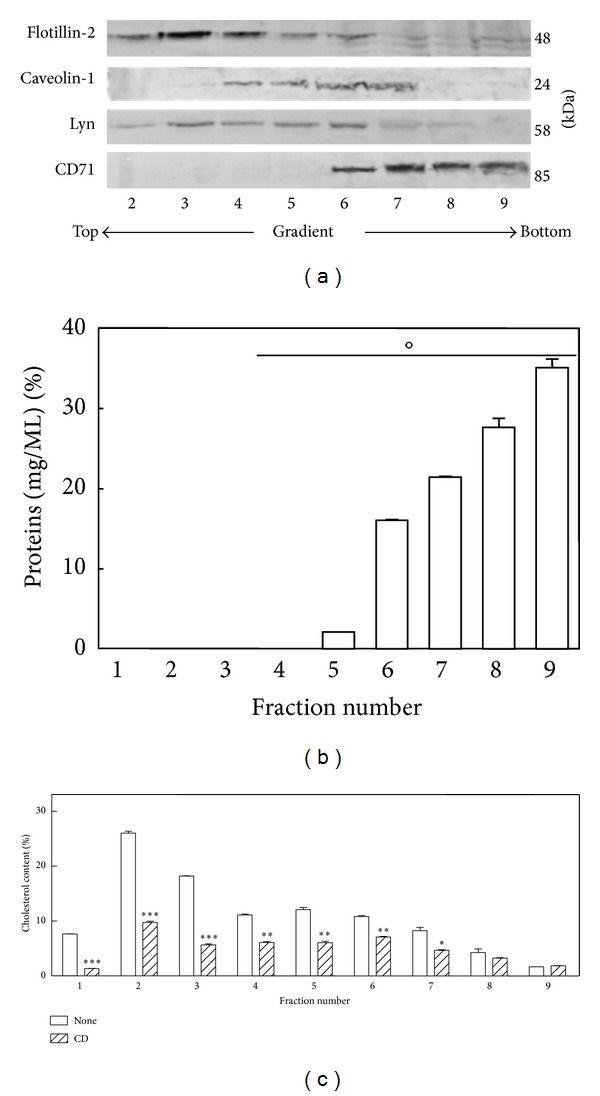
Isolation and identification of membrane caveolae/lipid rafts by detergent extraction and sucrose gradient centrifugation in B1647 cell line. (a) Cells were lysed with 1% Triton X-100 at 4°C and separated by sucrose density-gradient ultracentrifugation as described in Section 2. Equal aliquots of each fraction were subjected to SDS-PAGE and Western blotting. Flotillin-2, caveolin-1, and Lyn were used as markers for caveolae/lipid raft fractions and CD71 for nonlipid raft fractions. (b) Typical profile of protein concentrations in gradient fractions after ultracentrifugation. Protein content was determined as described in Section 2. (c) Cells were preincubated at 37°C for 16 hours with [^3^H]-cholesterol (0.1 *μ*Ci/mL) in cell culture medium then exposed (or not) to 10 mM CD for 20 min, lysed with 1% Triton X-100 at 4°C, and subjected to sucrose density gradient ultracentrifugation as previously described. [^3^H]-cholesterol content of each fraction collected was quantified by liquid scintillation counting. Results are expressed as means ± SD of three independent experiments, each performed in triplicate. °*P* < 0.01: significantly different from each other; *P < 0.01: significantly different from untreated cells; **P < 0.005: significantly different from untreated cells; ***P < 0.001: significantly different from untreated cells.

As reported in Figure 2(a), flotillin-2, Lyn, and Cav-1 are localized in the low-density region of the gradient (fractions 2–6), between approximately the 10% and 25% sucrose layers, where caveolae/lipid rafts are supposed to be; instead, CD71 is located in the fractions 6–9.

The protein content of the different gradient fractions is reported in Figure 2(b), showing that the bulk of B1647 proteins was found in the high-density region at the bottom of the sucrose gradient.

Moreover, since it has been shown that CD is capable of removing cholesterol from both raft and nonraft fractions [34], the effect of CD treatment on cholesterol distribution in sucrose gradient fractionation was investigated. B1647 cells were labeled with [^3^H]-cholesterol as described in Section 2. Cells were then exposed (or not) to 5 mM CD for 20 min, lysed with Triton X-100, and subjected to sucrose gradient centrifugation. As reported in Figure 2(c), fractions 2–6, where caveolae/lipid rafts are localized, exhibited the highest cholesterol content. Moreover, the cholesterol distribution profile of the samples treated with CD shows that, in our experimental conditions, fractions 2 and 3 exhibited a higher cholesterol depletion, evidencing a more efficient cholesterol removal from detergent resistant membranes, that is, caveolae/lipid rafts, compared to the other fractions.

### 3.3. Effect of Cholesterol Depletion from Plasma Membrane on VEGFR-2 Localization, ROS Generation, and Glucose Transport Activity in B1647 Cells

Since we previously demonstrated that a VEGF-mediated redox signaling pathway, creating a loop, is responsible for maintaining high intracellular ROS level, glucose uptake, and consequently B1647 cell proliferation [20, 21], we performed an experiment in order to understand the importance of caveolae/lipid rafts in the VEGFR-2 activation, stimulated by the autocrine VEGF production, characteristic of B1647 cells [19]. To investigate the functional role of caveolae/lipid rafts in the regulation of these activities, CD, a well-established cholesterol depleting reagent was used to cause their disruption.

B1647 cells, in the presence or absence of human serum (HS) and/or CD, were lysed by TX-100, centrifuged, and the supernatants were collected then separated by sucrose density-gradient (5–40%) centrifugation as described in Section 2. Nine fractions were collected and SDS-PAGE followed by Western blotting was performed to observe the localization of VEGFR-2.

**Figure 3:**
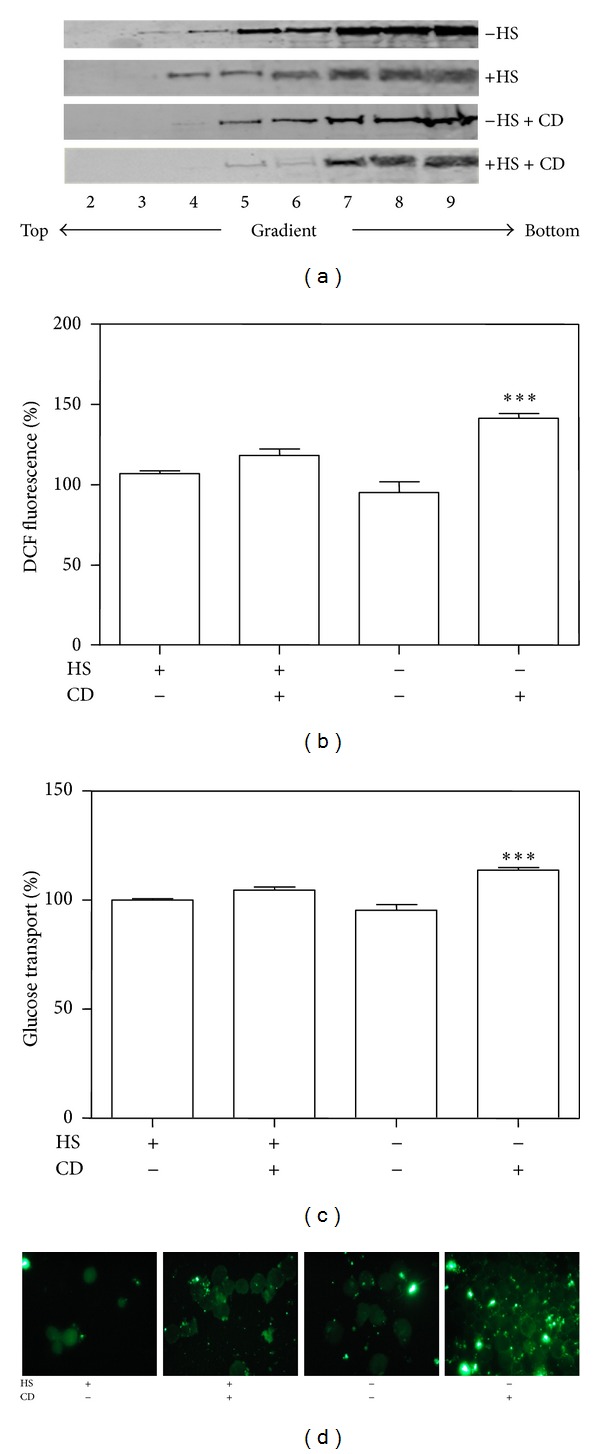
Effect of cholesterol depletion from plasma membrane on VEGFR-2 localization, ROS generation, and glucose transport activity in B1647 cells. (a) Cells in the presence or absence of human serum (HS) and/or pretreated with 5 mM CD for 20 min were lysed with 1% Triton X-100 at 4°C and separated by sucrose density-gradient ultracentrifugation as described in Section 2. Equal aliquots of each fraction were subjected to SDS-PAGE, Western blotting, and revealed for anti-VEGFR-2 (210 kDa). A representative blot is shown. (b) Cells in the presence or absence of human serum (HS) and/or pretreated with 5 mM CD for 20 min were incubated with 5 *μ*M DCFH-DA and ROS intracellular level was measured spectrofluorimetrically as described in Section 2. (c) Cells in the presence or absence of human serum (HS) and/or pretreated with 5 mM CD for 20 min were incubated with DOG mixture and glucose uptake was assayed by liquid scintillation counting as described in Section 2. (d) Cells in the presence or absence of human serum (HS) and incubated (or not) in PBS at 37°C with 5 mM CD for 20 min were fixed in 3% (w/v) paraformaldehyde for 15 min. Cells were then immunolabelled with anti-Glut1 (N-20) antibody (raised against an extracellular domain of Glut1, therefore, evidencing that Glut1 is present on the cell surface), treated with fluorescent FITC-conjugated secondary antibody, and visualized using immunofluorescence microscopy.

As reported in Figure 3(a), cell treatment with CD resulted in the redistribution of VEGFR-2 from caveolae/lipid rafts to noncaveolar fractions both in the presence and in the absence of HS. Moreover, the distribution of VEGFR-2 is slightly different between cells in the presence of HS or starved, suggesting that the VEGF autoproduced by cells in the presence of human serum (normal condition) is able to link to its receptor causing a shift of the VEGF-VEGFR-2 complex towards noncaveolar membrane regions.

Parallel experiments were performed in order to examine the effect of CD in the presence or absence of HS on intracellular ROS level by means of a fluorimetric analysis implying DCFH-DA (Figure 3(b)) and on glucose uptake by using ^3^H-DOG, a labeled glucose analogue (Figure 3(c)): both parameters increased after cholesterol depletion from plasma membrane by CD treatment, according to Western blotting results reported in Figure 3(a). These results suggest that VEGF-VEGFR-2 interaction, facilitated by the partial caveolae/lipid raft disruption, triggers a signal transduction pathway leading to an increase in ROS generation and glucose uptake.

Moreover, analyses of the images obtained by immunofluorescence microscopy of cells labelled with an anti-Glut1 antibody against an extracellular domain of Glut1 revealed that incubation with CD greatly enhances the staining for the transporter at the cell surface (Figure 3(d)). These results indicate that CD treatment increases glucose uptake through Glut1 recruitment into the plasma membrane from intracellular pool.

As previously shown [21], the absence of human serum caused a slight decrease both in DCF fluorescence and DOG uptake, indicating that VEGF self-production decreases in respect to normal conditions (i.e., in the presence of human serum) and/or that different agents modulate ROS production and glucose transport.

Thus, in order to better evaluate the role of VEGF, ruling out other effect due to other serum components, the following experiments were performed also on serum-depleted cells in the presence or absence of exogenous VEGF.

### 3.4. Association of VEGFR-2 with Caveolin-1

To better understand the relationship between caveolae/lipid rafts and VEGF receptor activation, the interaction/colocalization between VEGFR-2 and Cav-1 was investigated.

**Figure 4:**
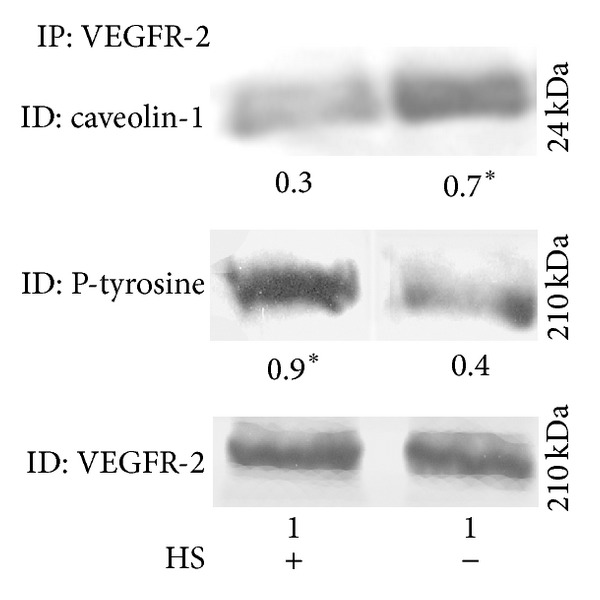
*Association of VEGFR-2 with caveolin-1 in B1647 cells. *Cells deprived or not of serum (±HS) were subjected to immunoprecipitation with anti-VEGFR-2 as described in Section 2. Samples were electrophoresed, immunoblotted, and revealed for anti-caveolin-1, for antiphosphotyrosine, or with anti-VEGFR-2. A representative blot is shown. Results were obtained considering three independent Western blot experiments. Relative amounts determined by scanning densitometry are expressed in arbitrary units. *P < 0.05: significantly different from control cells (+HS).

Therefore, B1647 cells were serum starved for 18 h and the colocalization of VEGFR-2 and Cav-1 was evaluated by immunoprecipitation experiments. As reported in Figure 4, in serum starved condition Cav-1 and VEGFR-2 colocalize and VEGFR-2 phosphorylation significantly decreases. These results indicate that the presence of VEGF could modulate the activation as well as the localization of VEGFR-2.

### 3.5. Importance of VEGF/VEGFR-2 Interaction for ROS Generation and Glucose Uptake

In order to corroborate the obtained results, we evaluated the effect of different compounds able to inhibit VEGF interaction with its receptor by different mechanisms.

In particular, B1647 cells in the presence or absence of serum (HS) were incubated for 2 hours with 3.4 nM Bevacizumab, a monoclonal anti-VEGF antibody [35], for 30 minutes or with 5 *μ*M Cav-1 scaffolding domain (CSD) that represents the essential portion (residues 82 to 101) for Cav-1 interaction with other proteins [36].

**Figure 5:**
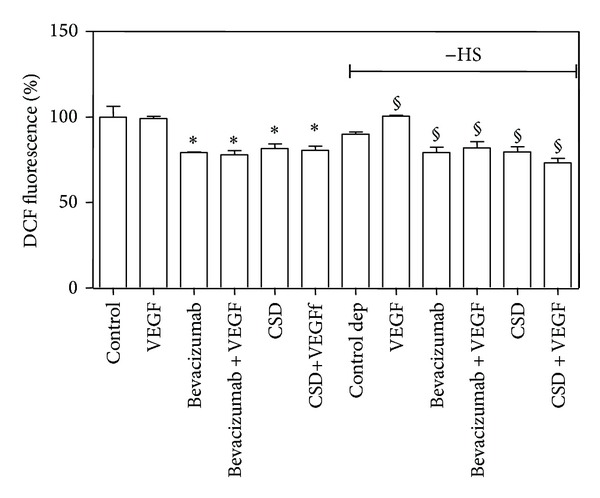
*Effect of different inhibitors of VEGF/VEGFR-2 interaction on intracellular ROS level in B1647 cells. *Cells, control, and serum starved (−HS, “Control dep”) were treated with 500 *μ*g/mL Bevacizumab for 30 min or 5 *μ*M Cav-1 scaffolding domain (CSD) for 2 h, in the presence or absence of 50 ng/mL VEGF; then, the intracellular ROS level was spectrofluorimetrically measured by means of DCFH-DA, as described in Section 2. *P < 0.05: significantly different from control cells; ^§^ *P* < 0.05: significantly different from serum deprived control cells (−HS).

Results showed that both Bevacizumab and CSD caused a decrease of intracellular ROS level in the presence or absence of HS (Figure 5).

50 ng/mL VEGF treatment, in the presence of inhibitors, was unable to induce ROS generation; on the contrary, it increased the intracellular ROS level in serum-depleted cells reaching the ROS content of control cells, confirming previously reported data [21].

**Figure 6:**
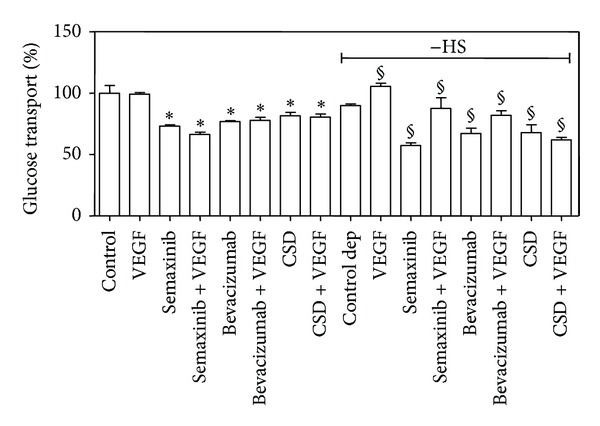
*Effect of different inhibitors of VEGF/VEGFR-2 interaction on glucose transport in B1647 cells. *Cells, control, and serum starved (−HS, “Control dep”) were treated with 20 *μ*M Semaxinib for 2 h, 500 *μ*g/mL Bevacizumab for 30 min, or 5 *μ*M Cav-1 scaffolding domain (CSD) for 2 h, in the presence or absence of 50 ng/mL VEGF; then, glucose uptake was assayed by liquid scintillation counting as described in Section 2. *P < 0.05: significantly different from control cells; ^§^ *P* < 0.05: significantly different from serum deprived control cells (−HS).

Subsequently, cells were subjected to the same treatments and analyzed for glucose transport, (Figure 6), obtaining results very similar to that reported in Figure 5.

Semaxinib (SU5416), one of the most frequently used inhibitor of VEGFR-tyrosine activity, was also utilized (20 *μ*M for 2 hours), obtaining a trend of response comparable to Bevacizumab and CSD effects. Semaxinib was not suitable for the detection of its effect on ROS intracellular level due to its autofluorescence [37].

Survival experiments performed by MTT assay demonstrated that cell viability did not change in these experimental conditions (data not shown).

### 3.6. Caveolin-1 Interaction with Nox2 and VEGFR-2 in the Presence or Absence of VEGF in B1647 Cells

It has already been reported that Nox2 is present in caveolae/lipid rafts in association with Cav-1 in vascular smooth muscle cells [38] and that Nox4 is mainly located in nonraft region of the plasma membrane [39].

**Figure 7:**
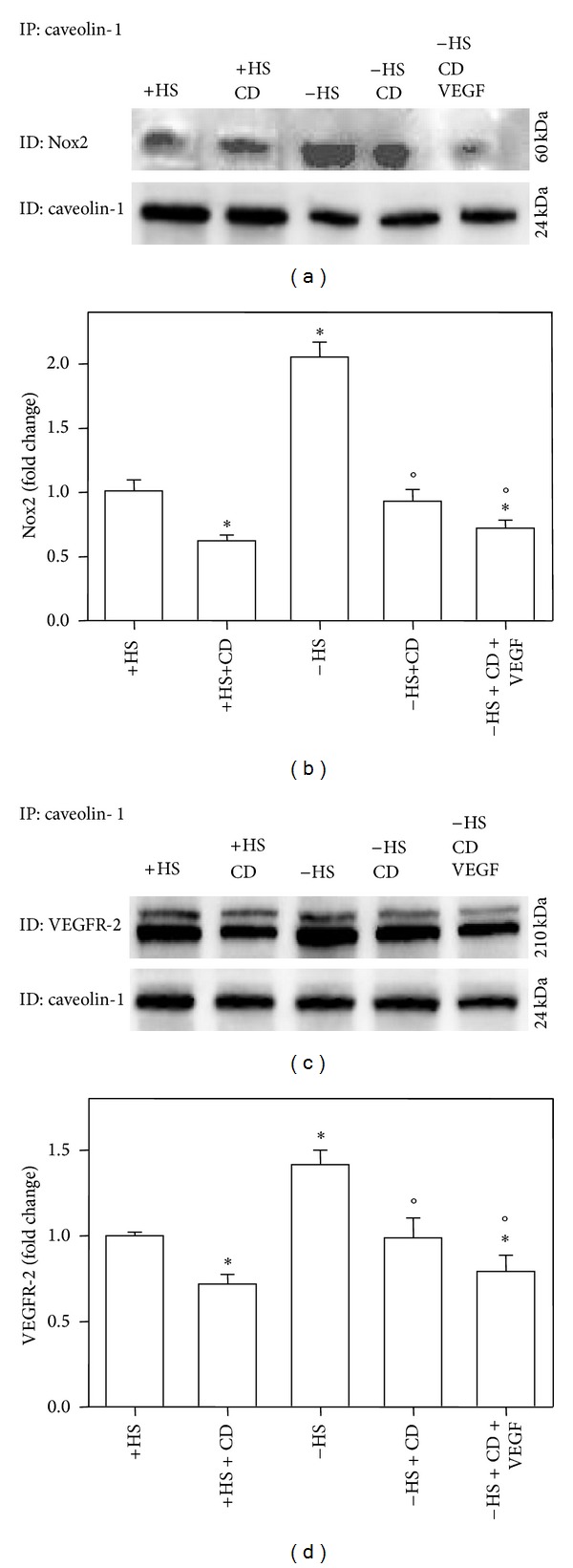
*Caveolin-1 interaction with Nox2 and VEGFR-2 in the presence or absence of VEGF in B1647 cells. *Cells deprived or not of serum (±HS) were subjected to immunoprecipitation with anti-caveolin-1 as described in Section 2. (a) Samples were electrophoresed, immunoblotted, and revealed for anti-Nox2 and anti-caveolin-1; a representative blot is shown. (b) Densitometric analysis normalized for caveolin-1 content and expressed as fold change in Nox2 expression with respect to control (+HS). (c) Samples were electrophoresed, immunoblotted, and revealed for anti-VEGFR-2 and anti-caveolin-1; a representative blot is shown. (d) Densitometric analysis normalized for caveolin-1 content and expressed as fold change in VEGFR-2 expression with respect to control (+HS). Results were obtained considering three independent Western blot experiments. *P < 0.05: significantly different from control cells (+HS); °*P* < 0.05: significantly different from control serum starved cells (**−**HS).

Figure 7(a) shows that in absence of VEGF (−HS) Nox2 colocalizes with Cav-1 in B1647 cells. CD and VEGF treatment significantly decreases Cav-1/Nox2 association, demonstrating that in absence of VEGF, Nox2 colocalizes with Cav-1, presumably into caveolae/lipid raft compartments.

After CD and VEGF treatment, VEGFR-2 association with Cav-1 significantly decreases and this effect is partially counteracted by HS starvation (absence of VEGF) (Figure 7(c)). Additionally, we did not observe a colocalization between p-VEGFR-2 and Cav-1 suggesting that the activation of VEGFR-2 occurs in noncaveolar compartments (data not shown).

## 4. Discussion

Caveolae/lipid rafts function as signaling organizing centers and platforms by exploiting multiple protein-lipid and protein-protein interactions to link the cytoplasmic tail of trans-membrane receptors with other protein scaffolds to assemble kinases, phosphatases, and other catalytically active molecules, consequently modulating specific signals that are temporally and spatially controlled [40, 41]. This concept is peculiar for redox signaling: in order to act as signal molecules, ROS must be generated in discrete compartments and following certain* stimuli* thus produced in a controlled manner from the standpoint of the space/time [42–44].

Regarding this context, we have been intrigued by recent reports showing the involvement of caveolae in the regulation of redox signal transduction mediated by VEGF in endothelial cells [10, 45–48]. VEGFR-2 signal transduction seems to follow the scheme for the activation of receptor tyrosine kinases, following receptor dimerization induced by VEGF binding. Dissociation of VEGFR-2 from caveolae has been shown to be essential for its autophosphorylation and activation of downstream signaling events [7, 47]. Moreover, VEGF and its receptors have been shown to be critical players in the embryonic development of endothelial and blood cells [49] and the evidence that angiogenesis plays a pathophysiological role in leukemia has been well documented [50].

Since endothelial cells share signal transduction pathways with hematopoietic cells [51], we investigated the potential role played by caveolae/lipid rafts in the modulation of redox signaling induced by VEGF, in a model of human acute leukemia: B1647 cell line.

We previously demonstrated that B1647 cells possess high level of Nox-derived ROS that sustain cellular growth and glucose uptake, creating a loop signal transduction suggested to be maintained by autocrine VEGF production [20, 21].

Frequently, leukemia cells contain relatively elevated intracellular ROS level and it has been previously established that Nox-generated ROS can trigger genomic instability and different downstream prosurvival pathways [52], even if the mechanisms involved are not completely understood. Their elucidation could have important therapeutic implications.

Several studies have shown that AML cells release angiogenic growth factors such as VEGF within the bone marrow and that subsets of acute leukemias also express VEGF receptors; thus, autocrine stimulation of leukemia cells by VEGF may result in proliferation, migration, and resistance to chemotherapy [53]; VEGF exerts indeed its trophic effect on malignant myeloid progenitors via either paracrine or autocrine interaction [54].

In the present work we report that VEGFR-2 is partially localized in caveolae/lipid rafts, and after the binding with VEGF a shift to nonraft portions of membrane occurs, causing the trigger of phosphorylation cascade by the activation of tyrosine kinase VEGFR-2 and leading to increase of glucose transport marker of cell proliferation.

The first step was the setting of the optimal conditions for obtaining a caveolae/lipid raft disruption by considerable cholesterol depletion from membrane without affecting cell viability during the time of the experiment. As tools to achieve this goal we used CD, reported to have the highest affinity for cholesterol inclusion and to be the most efficient in extracting cholesterol from membranes [22]; nevertheless, the CD efficiency may vary significantly depending on its concentration, duration of the exposure, and cell model. In our conditions, cell treatment with 5 mM CD for 20 min was able to remove 60% of cholesterol from plasma membrane without seriously affecting cell viability.

Because of the lack of a standardized method to detect and isolate caveolae/lipid rafts [43], here the experimental evidence of their existence is given by their resistance to solubilization by the nonionic detergent TX-100 1% at 4°C followed by density-gradient centrifugation [55]. In order to exclude the hypothesis of artifacts, we used an alternative method to isolate raft-like membranes with a detergent-free medium containing a high sodium carbonate concentration, obtaining similar results (data not shown).

Even if several studies revealed that *β*-cyclodextrins are capable of removing cholesterol from both raft and nonraft fractions [22], the efficiency of cholesterol removal preferentially from lipid rafts is obtained by using short time exposures or very low CD concentration [34]. Figure 2(c) demonstrated that although the cholesterol content of all membrane fractions was significantly reduced, in our experimental conditions CD was able to remove cholesterol more efficiently from detergent resistant membrane, representing the fractions containing caveolae/lipid rafts.

Our results show that in sucrose gradient fractionations of lysed B1647 cells (+HS), VEGFR-2 is present both in raft and nonraft regions. When cells are deprived of serum (−HS), VEGFR-2 content increases in the caveolae/raft fraction and when CD was added to the cells VEGFR-2 is mainly present in nonraft fraction. These results compared to data on the ROS intracellular level and glucose uptake (Figures 3(b) and 3(c)) of cells subjected to the same treatments (i.e., in the presence or absence of serum and in the presence or absence of CD) suggests that when VEGF, normally autoproduced by B1647 cells, binds to its receptor it causes its activation, displacing it from raft to nonraft regions. The disruption of rafts allows more interaction between VEGF and its VEGFR-2 receptor, increasing its phosphorylation and modulating the derived signal transduction pathways leading to glucose uptake. Images reported in Figure 3(d) confirm the increase in Glut1 isoform on the plasma membrane of cells in the presence of CD.

Moreover, we demonstrated that VEGFR-2 in the absence of serum is more linked to Cav-1, a major caveolae component [7]; instead, in normal condition (+HS) where VEGF autocrine production is* bona fide* high, VEGFR-2 binding to Cav-1 significantly decreases promoting its activation, as demonstrated by the higher level of phosphorylation observed. These data are in accordance with those obtained in endothelial cells, where Cav-1 acts as negative regulator of VEGFR-2 activity [7].

To deeply analyze the importance of VEGF/VEGFR-2 interaction to induce ROS production and related glucose uptake [21] and the downregulation exerted by VEGFR-2 localization in caveolae/lipid rafts, we tested, in control and serum starved cells, different compounds able to inhibit the binding of VEGF to its receptors. In particular, Bevacizumab was selected as a monoclonal anti-VEGF antibody highly active against several cancers [56]. Today, it is the most commonly used anti-VEGF drug against tumor-derived VEGF [35, 57], validating Folkman's early prediction on the importance of inhibit tumor angiogenesis [58]. Nevertheless, the wide adverse effects induced by anti-VEGF agents demonstrate that these drugs have a broad impact on vasculatures in multiple healthy tissues and organs.

As previously cited, caveolae are coated with a 24 kDa protein, Cav-1. This protein regulates multiple cancer-associated processes including cellular transformation, tumor growth, cell migration and metastasis, cell death and survival, multidrug resistance, and angiogenesis. However, Cav-1 has been reported to influence both positively and negatively various aspects of tumor progression and to act as tumor suppressor or poor prognostic factor in many human cancers [8].

To clarify the role of Cav-1 in our model, we incubated the cells with the Cav-1 scaffolding domain (CSD), representing the portion of the protein (residues 82 to 101) essential for both Cav-1 oligomerization and the interaction with other proteins [36]. Associations with other proteins through the CSD provide coordinated and efficient signal transduction [59]. CSD binds many signaling molecules, including endothelial nitric-oxide synthase (eNOS), Src-like kinases, Ha-Ras, and heterotrimeric G-proteins [60]. Binding of these proteins to CSD in many cases negatively regulates their function [61]. Moreover, acute vascular inflammation in mice was prevented by systemic administration of cell-permeable CSD peptide [62].

Our results suggest that Cav-1 or VEGFR-2 localization in caveolae may act as negative regulators of the receptor activity. Similarly, the treatment of HUVEC with CSD caused significant reduction in the VEGF-stimulated phosphorylation of VEGFR-2, suggesting that CSD inhibits Cav-1- mediated angiogenic signaling [47].

Last but not least, Semaxinib, a reversible, ATP-competitive, oxindole-based inhibitor of VEGFR-2 tyrosine kinase, inhibits VEGF-dependent phosphorylation of the VEGFR-2 overexpressed in NIH 3T3 cells with an IC_50_ of 1.04 *μ*M. In an ELISA-based assay, Semaxinib inhibits autophosphorylation of the VEGFR-2 at an IC_50_ of 1.23 *μ*M [63]. The results obtained with the three compounds able to inhibit VEGF/VEGFR-2 interaction confirm the importance of this molecular complex in maintaining the redox loop leading to high intracellular ROS level and glucose uptake of B1647 cells.

We previously reported that, in B1647 cells expressing Nox2 and Nox4, VEGF signaling and Nox activity are coupled [20, 21]; moreover, it has been recently demonstrated that inhibitors of both Nox and VEGF receptors are able to induce apoptosis in leukemia cell lines and that lipid rafts play a role in this process [40]. Thus, we performed experiments to evaluate the involvement of caveolae/lipid rafts in the coordination of VEGFR-2 activation and ROS production by Nox2. Nox4, indeed, is a constitutive active isoform [64] reported to be mainly present in nonraft region of the plasma membrane [39].  Figure 7 suggests that VEGF promotes the dissociation of both Nox2 and VEGFR-2 from Cav-1 and the consequently activation of Nox2–VEGFR-2 axis in nonraft fractions. Our results are in accordance with those by Han and colleagues [65], demonstrating that in human renal proximal tubule cells the majority of p22phox and Rac1 is distributed in lipid rafts, whereas Nox4 is excluded from them. Cholesterol depletion increased NAD(P)H oxidase activity by redistributing NAD(P)H oxidase subunits in nonlipid raft membrane, suggesting that in human nonphagocytic cells, lipid rafts keep NAD(P)H oxidase (Nox2) in the inactive state.

Our data support recent findings reporting that the recruitment of specific receptors, transporters, and isoforms of NAD(P)H oxidase within membrane microdomains generates redox signaling platforms, recently defined as “redoxosomes,” which could be the missing link between receptor activation and enzymatic generation of ROS [48, 66].

Moreover, results here reported suggest emerging targets for new pharmaceutical application and clinical translation.

## 5. Conclusions

In this study we evaluated the potential involvement of caveolae/lipid rafts in the modulation of VEGF-induced redox signal transduction in leukemia cells.

We demonstrated, for the first time to our knowledge, that the colocalization of VEGFR-2 and Nox2 in caveolae/lipid rafts is involved in the negative modulation of glucose uptake, necessary to the deregulated proliferation of B1647 leukemia cell line.

Studies of how the redox system is controlled and balanced towards/against a proliferative advantage in leukemia cells suggest new therapeutic targets and may hold the key to unlocking therapeutic resistance in leukemia.

## Abbreviations

AML: Acute myeloid leukemia
B1647: Human erythromegakaryocytic leukemia cell line
Cav-1: Caveolin-1
CD: Methyl-*β*-cyclodextrin
CD71: Transferrin receptor
CSD: Cav-1 scaffolding domain
DCF: Dichlorofluorescein
DCFH-DA: Dichlorofluorescin diacetate
DOG: 2-deoxy-D-glucose
DPI: Diphenyleneiodonium chloride
Glut1: Glucose transporter-1
HS: Human serum
Nox: NAD(P)H oxidase
ROS: Reactive oxygen species
VEGF: Vascular endothelial cell growth factor
VEGFR-1: Vascular endothelial growth factor receptor-1
VEGFR-2: Vascular endothelial growth factor receptor-2 (also known as KDR).
